# “It is in the Nature of Men”: The Normalization of Non-Consensual Sex and Intimate Partner Violence Against Women with Acquired Physical Disabilities in South Africa

**DOI:** 10.1177/10778012231172710

**Published:** 2023-05-02

**Authors:** Xanthe Hunt, Amelia van der Merwe, Leslie Swartz, Wendy Xakayi, Yeukai Chideya, Laura Hartmann, Michelle Botha, Alison Hamilton

**Affiliations:** 1 Institute for Life Course Health Research, Department of Global Health, 26697 Stellenbosch University, Cape Town, South Africa; 2 Department of Psychology, 26697 Stellenbosch University, Stellenbosch, South Africa; 3 Department of Psychiatry and Biobehavioral Sciences, University of California Los Angeles, Los Angeles, CA, USA; 4 VA Center for the Study of Healthcare Innovation, Implementation, and Policy, VA Greater Los Angeles Healthcare System, Los Angeles, CA, USA

**Keywords:** disability, intimate partner violence, gender

## Abstract

This study employed a cross-sectional, qualitative individual interview methodology to explore South African women with physical disabilities’ experiences of intimate partner and sexual violence, inclusive of non-consensual and coerced sexual intercourse. For the participants, disability was a factor that intersected with gender norms to create vulnerability to abuse, and that patriarchal ideologies constructing how women should perform their gendered roles in marriage or sexual partnerships, as well as disability stigma, exacerbated this vulnerability. It is important to develop understandings of the different risk factors for violence – at the individual level and in the context of dyadic relationships – to develop programming to better support women.

## Background

South Africa has strikingly high rates of violence against women. It has been identified as having the greatest prevalence of rape for any country not engaged in war (du Toit, 2014), and each year, the South African Police Service file reports on close to 65,000 rape cases with an estimated one million rapes going unreported (du Toit, 2014). Every six hours, a woman is killed by an intimate partner in South Africa, resulting in the world's highest reported rate of intimate femicide – more than five times the global average (Mathews et al., 2004). In 2016, 17,250 out of every 100,000 women were victims of sexual offences (StatsSA, 2018), and in 2021, over the course of just three months, 9556 rapes were reported to police (Ndenze, 2021). The lifetime prevalence of sexual and/or physical intimate partner violence (IPV) among women is between 40% and 55.5%, and rape is between 12% and 28% (Dunkle, 2004; Jewkes, 2002; Jewkes et al., 2010; Jewkes & Abrahams, 2002).

As much as being a woman in South Africa is associated with risk of exposure to violence, so is being a person with a disability. Research has shown that people with disabilities are more likely to be victims of violence than people without disabilities (Hahn et al., 2014; Stern et al., 2020). Experiences of violence are particularly common amongst women with a disability, with these women facing more incidents of gender-based violence (GBV) and IPV than women without disabilities (Cohen et al., 2005; Hahn et al., 2014; Khalifeh et al., 2015; Martinet & Legry, 2014; Schröttle & Glammeier, 2013; Stern et al., 2020). The prevalence of disability-related violence against women in South Africa has been linked to greater disability-related stigma, poor access to health and support services, lack of resources for people with disabilities, and lack of public education on disability and disability rights (Mall & Swartz, 2011; Mavuso & Maharaj, 2015; Van der Heijden et al., 2019).

The association between disability and violence is explained, at least in part, by several factors. Firstly, people with disabilities may be more dependent on others (and thus more vulnerable to others). They are also, on average, poorer than people without disabilities, and poverty is associated with increased risk of violence. For instance, living in poverty is associated with higher rates of violence against women (Barrnet et al., 2010). Economic disadvantage also increases the likelihood of entering and remaining in abusive relationships (Nosek et al., 2001). Finally, people with disabilities’ risk for isolation related to a lack of accessible environments and functional impairments (and thus difficulty in accessing help), as well as prevalent problematic societal attitudes towards disability, exacerbate vulnerability to violence (Hahn et al., 2014). Evidence also suggests that women with disabilities are unlikely to seek help when they are victimized due to fear of retaliation from perpetrators, having their credibility as reporters of violence questioned, or being dismissed by service providers (Barrett & Pierre, 2011). Research conducted in South Africa has shown that stigma related to disability is a key obstacle in women with disabilities reporting experiences of GBV (Van der Heijden et al., 2019). For example, the testimonies of women with intellectual disabilities in court cases are often disregarded if the women's speech is impaired or inconsistent (Martinet & Legry, 2014).

In South Africa, as in many countries, part of the reason for high rates of violence against women are social norms and values which normalize violence generally, and sexual violence and violence against women, particularly. Violence against women in the South African context is perpetuated by widely held, false beliefs about women and gender norms in relationships (Zembe et al., 2015). Some research, for instance, suggests that men in South Africa hold the misconception that physical violence is a necessary expression of love, normal in relationships between male and female partners (Zembe et al., 2015). Conversely, young women may believe that violent men are desirable partners and “real men” (Zembe et al., 2015). IPV may, therefore, be normalized and seen as a necessary component of the power and intimacy dynamic in a sexual relationship (Zembe et al., 2015).

The triple vulnerability of women with disabilities (based on gender, disability status, and socioeconomic status) to violence makes the foregrounding of the rights and needs of women with disabilities in GBV prevention efforts and response services all the more important (Van der Heijden et al., 2019). It is against this backdrop that the present paper explores women's experiences of IPV and sexual violence in the context of a larger study on the sexual and reproductive health of women with road accident acquired physical disabilities.

## Method

This study employed cross-sectional, qualitative individual interview methodology.

### Setting

The study drew participants from Khayelitsha, a peri-urban area near Cape Town, South Africa, with many informal dwellings and an estimated population of between 500,000 and 750,000 people. Extrapolating from samples in neighbouring areas, the disability prevalence is about 13.1% (Maart, 2015).

### Sampling and Recruitment

Flyers advertising the study were distributed in the area surrounding the first author's research unit, and participants were recruited through snowball sampling. Specifically, a staff member working at a community research centre in the study site accessed informal social groups for women with physical disabilities as well as other networks known to her in the area. Women were eligible for inclusion in the study if they responded to the advertisement and:

1. Had a physical disability which was acquired as a result of a motor-vehicle accident;
2. Were over the age of 18 years;
3. Identified as a woman;
4. Resided in the Western Cape; and
5. Were are able and willing to give informed consent to participate in the study.

### Participants and Procedure

All data collectors were employed by the same research unit as the first author, in Khayelitsha. These data collectors were trained by the first author in qualitative interviewing principles, disability, and sexual and reproductive health. Most of the research assistants had previous experience of conducting interviews, and, crucially, were all first language speakers of the preferred language of the participants to whom they were assigned (isiXhosa).

Women who responded to the advert were screened over the phone by the research unit's community centre coordinator to assess whether they met inclusion criteria, and those who met the inclusion criteria were asked whether they would like to participate in the study. After the participants were screened, the centre manager discussed the study information sheet with them. The prospective participants were informed that the interview would take approximately an hour and a half to complete, and appointments were scheduled to obtain consent.

During the in-person appointment, the study information sheet was discussed in detail once again. The data collector then obtained informed consent, after either reading the form to the participant or the participant reading it herself, depending on literacy levels. Once informed consent was obtained, the data collector conducted the interview in the home language of the participant (isiXhosa). All interviews were audio-recorded and checked for content and quality, and the audio-recording was transcribed and translated into English (and back-translated) by trained transcribers and translators. Once the interview was completed, participants were given a R140 (approximately $10) gift voucher to a supermarket, Checkers, to thank them for contributing their time to the study.

### Materials

The interview guide consisted of questions focused on participants’ experience of the acquisition of disability, sexual relationships, sexual and reproductive health services, and health services’ access generally. The full interview schedule is available from the authors upon request, but included such items as:

1. Have you been in a sexual relationship since the accident? And, if yes, has it been any different to relationships before the accident? *If you have not, do you mind me asking why not?*
2. Do you feel like men (or, if you date women, women) have ever treated you differently in dating or romantic settings because of the fact that you are a woman with a physical disability?
3. Since acquiring your disability, have you ever experienced any form of sexual violence? This includes things like being raped, or being sexually assaulted, or having someone do something sexual to you that you did not want? What happened here, and what was this experience like? *Did you ever seek help about this?*
4. Since acquiring your disability, have you ever experienced any kind of intimate partner violence? This includes someone being emotionally abusive (shouting at you a lot, making you scared or threatening you) as well as someone physically hurting you (for instance, kicking you, hitting you, or otherwise harming you physically)? What happened here, and what was this experience like? *Did you ever seek help about this?*
5. Since acquiring a disability, have you ever felt that you had to stay with a partner that you would prefer not to have stayed with, because you felt financially dependent on them? What happened here, and what was this experience like? *Did you ever seek help about this?*

The focus of this article is on experiences of violence in sexual relationships.

### Ethics

Ethical approval was given by Stellenbosch University's Health Research Ethics Committee (HREC), approval number N19/03/037. Participants were assured that their participation was voluntary, and informed that they may decline to participate or withdraw participation at any time without any adverse consequences. Written informed consent was obtained from all participants. Participants’ responses to the questions in the interview schedule were kept confidential.

### Analysis

Only interviews including accounts of violence experiences were included in the present analysis. Multiple case study analysis was used to analyze the data (Stake, 2015). This method was selected as it can assist in obtaining a textured understanding of personal narratives while allowing for cross-cutting themes to be identified.

All translated transcripts were read and the cases involving violence identified. These cases were then double coded for key themes and narrative patterns; first by the PI, and then, independently, by a research assistant who was a first language isiXhosa speaker. This was particularly important because isiXhosa is rich in metaphor and idiom, which can get lost in translation to English. A particular procedure was followed: the isiXhosa-speaking coder would refer back to the data collector who interviewed a given woman, or speak to the transcriber who was involved with the transcript in question, to establish what the isiXhosa referent for a specific translation was, and notes would be added in the transcripts to clarify the meaning of any language-specific metaphors or idioms. This role, which is called cultural brokering in the literature about language interpreters, is important to safeguard the lifeworlds of the participants, and to ensure that meanings are accurately conveyed (Gustafsson et al., 2013; Kilian et al., 2020). Furthermore, the use of independent coders and oversight researchers is essential to enhance reliability and validity in qualitative analysis (de Wet et al., 2020).

## Results

Of the 18 women interviewed for the parent study, four (Participant 1, Participant 2, Participant 12, and Participant 13) described having ever been the victim of violence or abuse in the context of intimate relationships, while two women (Participant 6 and Participant 16) provided data relevant to the topic, but not personal disclosures of victimization. Each participant's experience of IPV (each case) is summarized in a narrative extract below, with attention paid to emerging meanings in the data. Exploration of cross-cutting thematic findings is presented in the Discussion which follows.

Participant 1 is a 40-year-old who acquired a physical disability at age 31. She described coerced sex at the hands of a partner with whom she had an existing relationship at the time of the accident, but who only began to victimize her after she acquired a disability. She recounted:

I can say there [was] a slight difference [in our relationship before and after the accident] because there were times when he wanted us to meet and have sex and that time I told myself that I am not right: maybe I have [a] painful waist, I have back pains, I am not in the mood for what he wants us to be in the mood for, but he stops [or] sometimes he insists, sometimes he stops and listen to what I am saying.

Interviewer: How do you feel when he insists?

On that one I become wrong (not well) I say to myself he is taking advantage because I am like this. If I was not on [a] wheelchair maybe he was going to do what I want him to do. [After the accident] he takes advantage.

She added:

After it has happened [I raise it with him], but I wait. I don’t raise it the same day. If it happened tonight, I will wait then the following day. I would tell him look I didn’t like what happened and how it happened. Men always tell themselves that I am the man, when I need to get something I must get it, I am a man, I couldn’t control myself that time.

Interviewer: Then he would apologize?

He would then apologize, however the apology it is pointless because he would do it again on another day or month; do the same thing he apologized for.

She went on to normalize her experience by saying:

I can say…it is in the nature of men because even when we are having a conversation as women you would find that [other women's partners’] is doing as your partner.

Interviewer: Okay, so can you say …

Whether we are disabled or not disabled [sic].

Interviewer: So that is something done by men to women?

I can say so.

Participant 1's interview ended with a turn in which, contrary to the experiences she shared (above), she indicated that she was treated well (discussed further below):

Interviewer: Sometimes people with disability have good and great experience in a relationship, and sometimes they have bad and not great experiences. Do you think male partners have treated you differently in a relationship because you are a woman with a physical disability?

You see with the one I am with now, even with the one I was seeing before, I haven’t been treated bad, I was never treated bad.

Interviewer: Okay, okay you were never treated badly, you are treated well?

Yes.

Participant 2 is a 28-year-old woman who acquired a disability at age 19. She described abuse by her husband to whom she had been married at the time of the accident, but who only began to behave violently towards her after she acquired a disability. She described the changes which happened in their relationship following her acquiring a disability:

Before I got in an accident it was fun, but after I got in an accident this changed.

Interviewer: How did it change, my sister?

I didn’t get the love the way it was, so I can say that I was just a useless person…

Interviewer: That is what he was saying?

Yes, and there is no one else that can fall in love with me again.

Interviewer: He put it that way?

Yes, he put it that way, so things changed in that way.

This participant described the following:

He was talking things that were making me angry.

Interviewer: And also beating you?

And there was also a child in all that, as he were doing those things the child was also crying that side.

Interviewer: The child was still young?

Young, if he went out during the day, he would come back around 4 am telling me to talk, but not knowing what to talk about. I would tell that I would never be able to talk this time, but let us talk in the morning when you not drunk.

Interviewer: And then what was he going to do in the morning as you spare that time for him to talk?

He refused to talk that time. [When he did talk], I ignored him but then I became angry, I hold his t-shirt roughly and it teared, he hold the night dress I was wearing, and he teared it up to here, then he started insulting me about everything, about my home, myself, telling me that I’m useless, what she mentioned…[thinking…] said I’m like a dead person even though I’m alive, things like that.

She left him and eventually requested a formal divorce, but noted to the interviewer that this happened only because she felt she had no other way to escape him, as restraining orders were ineffective in stopping the abuse:

Mm… I do have some protection orders, but those protection orders never helped because I was calling the police when he tries to do his silly things, but all those things never helped me, even social workers were aware about what [was happening].

Interviewer: Social workers was…

He was just arrested but then come back, just be arrested, come back and start again where he left off, so I decided that instead of always opening a case against him, let me just leave him.

She concluded her interview by reflecting on the effect which these experiences have had on her relationships and dating experiences since acquiring a disability:

Interviewer: What is the reason for you not to date?

Because I’m in the wheelchair, even if someone tells me that he loves me, I don’t trust how true is that. I don’t trust them and I don’t even trust them when they say they love me, I don’t trust them. I have that mentality of I’m in a wheelchair, how can he love more though there are good people walking with two feet, I’m in a wheelchair with unmoving feet, with a changed shape, then why he loves me while there are people, so it is when I tell myself that they play with me.

Participant 13 was a middle-aged woman who did not disclose her exact age, who acquired a disability during a car accident in 2017. She also described being in a relationship which had started prior to her accident, but which only turned violent following her injury. She recounted:

[Before and in the direct aftermath of the accident] He was doing things for me sister. He would make me some tea, prepare me some porridge, and give me water to bath, and I would bath when I am at his place not at home.

Interviewer: When was that?

He would cook for me, so this bad behaviour is new, it is new. I think it was in May or February or in March [of the year following the accident] then he started beating me up.

She added, later in the interview:

Yes, he was beating me and I don’t even know the reason.

Interviewer: When did he start beating you?

He would just beat me.

Interviewer: Was he beating you before the accident?

No, he was not beating me, we were only arguing, he never beat me.

Interviewer: How many times did he beat you, do you remember how many times?

Now.

Interviewer: Yes

He was beating me all the time.

Interviewer: All the time?

All the time.

Participant 12 is a 21-year-old who acquired a disability at age 19. She had not had experiences of violence or abuse in the context of intimate relationships prior to acquiring a disability (but due to her age had not dated extensively). However, since dating as a woman with a disability, she has experienced emotional abuse at the hands of a romantic partner. She framed her experience by explaining, at the beginning of the interview, that she does not enjoy sexual activity as she does not like to be seen naked by men, but that she engages in it with her partner for fear that he will leave her or cheat on her if she does not:

Maybe… I’m the one who do not expose her body and then I don’t like sex at all. When it is time to have sex, it is obvious that you have to take off your clothes. This boyfriend of mine he likes sex, then I do it just to satisfy him since he is my boyfriend, [and] because maybe he will go to others. I just do it since he is my boyfriend, but deep down in my heart I don’t engage with it.

As noted, two other participants, while not explicitly reporting experiencing emotional or physical abuse in relationships, made comments relevant to the analysis here. One woman, 39-year-old Participant 16, described emotionally coerced sex in the context of her marriage, but contradicted herself by saying that although she felt coerced, she “was not forced”.

There is a bit of a difference [in our relationship since I acquired a disability]. It is not the same as before; now sometimes I feel like my husband is abusing me because to me I would like to have it when I like, because if I don’t like, I don’t even have those feelings.

Interviewer: You mean sex?

Yes.

Interviewer: Okay, so sometimes he wants while you are not in the mood?

Yes, but he doesn’t force me. He would say, ‘If you don’t want it is fine, I don’t want you to feel the pain.’

Participant 6, a 67-year-old woman who acquired a disability in her middle age, legitimized poor treatment of women with disabilities, by explaining that some of the accommodations necessary to live with a disability made women ‘undesirable’.

They are not taking care while they live with men in the houses, you would find that they smell bad.

Interviewer: Mm.

I say to them when people come, they should not come and find the bucket that you pee on under the bed, when people come, it should be clean, and you should also be clean.

Interviewer: Mm.

One is supposed to wake up and bath, put on makeup and not expect people to come and be the ones that get rid if your urine from last night.

## Discussion

It is important to note at the outset of our discussion that in this study, the numbers are small and our sample not necessarily representative. The case data we have cannot definitively answer the question of whether disability is an added risk factor for IPV or other forms violence. The emotive and highly suggestive findings we have, however, underscore the importance of exploring this issue further using a range of methods.

Of the four women who had experienced physical violence in relationships, three reported that the violence began only after they acquired a disability, a finding which would be consistent with the view expressed in the literature that there may be increased vulnerability associated with the disability. Men's perceptions of bodily change or functioning may catalyze or trigger violence in intimate partners). As we have noted, in the broader disability literature, there is evidence of elevated rates of violence against people with disabilities (Hassouneh-Phillips & Curry, 2002; Lee et al., 2015; Rich, 2014), however there is not much evidence specifically concerning violence exposure beginning after acquiring an impairment, clearly an area for broader and more systematic study.

In the stories we heard, a key underlying theme was that of power. Power relations within an existing relationship may change because of a partner acquiring a disability, or existing power imbalances may be exacerbated. Abuse of people with disabilities is often connected to unequal power relations between people with disabilities and people without disabilities, where people with disabilities are perceived as ‘less than’ people without disabilities (Krnjacki et al., 2015). Many people with disabilities have greater dependence on caregivers than other individuals of a similar age (Thiara et al., 2011).

Disability may also affect a person's ability to protect themselves (physically or psychologically), the likelihood of reporting the violence, and escape from a partner or seek help (Nosek et al., 2001). In the context of an existing relationship, the male partners’ perceptions of women ‘losing status’ due to their impairment, may have elicited a sense of impunity with respect to their behaviour, making possible actions which they may not have previously considered. This idea, that disability renders women lesser to women without disabilities (and, presumably, men), is supported by, for instance, Participant 2's account of being told she is “like a dead person” and “just a useless person”.

Where women were engaging in new relationships since acquiring a disability (such as Participant 12), and began experiencing violence in the context of these, offender related factors may offer additional layers of explanation. In these cases, the male partners began their relationship with the women in the context of their disabilities, and so their violent actions may be more reflective of patriarchal values and a choice to target women with disabilities due to a need to assert control over women or those perceived to be ‘weaker’ than themselves (Petersilia et al., 2001). Although not widely documented, some men without disabilities experience sexual attraction to people with disabilities (Solvang, 2007), which may in some cases represent an attraction to vulnerability and the potential it holds for exploitation and violence. In some cases, perpetrators may respond to perceptions of vulnerability with sexual aggression or physical violence. However, it is important not to pathologize sexual attraction to people with a range of body types, including bodies with impairments, and we are not suggesting that all or most men who become attracted to women with physical disabilities experience this attraction based on the possibility of enacting control or violence. Nonetheless, in the context of harmful, ableist social norms, such factors as domination and control may play a role in the violent behaviour described by participants.

In this regard, it is important to recognise that deeply psychically embedded ideas about disability, as explored by, among others, Campbell (2009), Hughes (2020) and Watermeyer (2014) potentially underlie the violent relationships described by the women in this study. Hughes (2020), for example, suggests that disabled people have long been held up as the social invalids against which ‘normalcy’, and social validity, is defined, measured and assured. We see, for instance, that – among our participants – these notions have been internalized in the form of potent self-stigma. Participant 2, for instance, described distrusting that anyone could love her, and stated that she has

that mentality of ‘I’m in a wheelchair, how can he love more though there are good people walking with two feet, I’m in a wheelchair with unmoving feet, with a changed shape, then why he loves me while there are people’, so it is when I tell myself that they play with me.

In light of this, we should consider a layer of experience, alongside the material circumstances described here, at the psychic level, that might influence the violent behaviour of men, themselves potentially socially invalidated by poverty. Thinking about the unconscious material related to disability invalidation that may underpin violence towards women with disabilities broadens our insight into the complexity of these relationships, and the psychic pay-offs that men are potentially gaining through perpetrating violence against their disabled partners.

Feminist disability scholars draw attention to the role of ableist attitudes and patriarchal domination in explaining risk factors for violence against women with disabilities (Brownridge, 2006). Brownridge (2006) argues that women with disabilities may be viewed by patriarchal men as less challenging to dominate, and these attempts to dominate may include violence. Violence may also be motivated by a desire to gain control over another, with evolutionary psychology emphasizing patriarchal men's need to exert and maintain control over ‘their sexual property’ (Wilson & Daly, 1998). The cultural devaluation of women with disabilities leads to the attribution of stereotypic vulnerabilities, including that woman with disabilities are “asexual, passive, unaware, and therefore, easy prey” (Nosek et al., 2001, p. 178). Substance abuse is another important perpetrator-related risk factor for violence against women with disabilities, but this was not mentioned in our interviews and warrants further investigation. It is possible that perpetrators who are also caregivers may experience dependency stress, and this may be linked to violence, which can be exacerbated by alcohol consumption in particular (Petersilia, 2001). It is important to develop understandings of the different risk factors for violence – at the individual level and in the context of dyadic relationships – in order to develop programming to better support women.

As emerged in our data, many women who have experienced violence also normalize the behaviour. This normalization has the effect of making the dataset conflicting, as on first reading it was easy to code a particular woman as having experienced abuse, and as simultaneously not having experienced abuse - her narrative would clearly include an instance of abuse, but when asked explicitly, she would state that she had not been abused. Even extremely brutal acts of violence were minimized or negated. This normalization may be for a range of reasons, one of which is that women who live with activity limitations are dependent on others for the accomplishment of daily tasks and may perceive the abusive partner to be her only caregiving option, and that the violence she is exposed to is the price she has to pay for survival. It could also be the case that in the context of South Africa's high rates of intimate partner violence, these experiences are normalized in relationships broadly.

Nonetheless, from our small data set, it appears that women's risk of sexual violence may be exacerbated by the denial of the desirability and sexuality of women with disabilities. These women may have fewer opportunities to learn about their sexuality through limited exposure to education and due to frequent rejection or overprotection (Womendez & Schneiderman, 1991) – possibilities which are supported by other findings from the larger project in which this data is embedded (Hartmann et al., 2022) and other project conducted by some of the authors in South Africa (Hunt et al., 2021). This may leave women with disabilities believing that celibacy or violent sexual experiences are their only choices, which is compounded by the belief that no good or loving person will ever be attracted to them (as seen with Participant 2's case) (Nosek et al., 2001). Some may believe that abusive and painful experiences are better than none (Nosek et al., 2001).

Other factors which may influence an individual's willingness to name violence experiences as such is that some people may view abuse as a normal occurrence because they had seen similar things happen within their family structure (Curry et al., 2011; Decker et al., 2013; Hamilton & Goeders, 2010; Othman et al., 2013), or may convince themselves that their partner's abusive behaviour may only be temporary and will change eventually (Curry et al., 2011; Othman et al., 2013).

Well-documented social norms – often common to a range of cultures and societies – act as barriers to discussing or even admitting to experiencing abuse (Curry et al., 2011; Othman et al., 2013). One such value is that family matters, including domestic violence, are to be kept within the family and kept secret from others (Decker et al., 2013; Lea & Lynn, 2012; Othman et al., 2013). This can even stretch as far as the belief that all issues within a marriage (even abuse) should not be shared amongst family members but rather solved between the couple (Byrskog et al., 2014; Decker et al., 2013; Lea & Lynn, 2012; Othman et al., 2013). The idea of ‘saving face’ is emphasized in a number of communities and as a result, women face pressure to not to ‘admit’ to being victimized in order to protect the reputation of their partner (Alaggia & Wang, 2020; Othman et al., 2013). Women may thus minimise their experiences in-keeping with social norms which frame the behaviour of their violent partners as dismissible. This may be even more of an issue for women with disabilities who risk social exclusion and being seen as non-normative on the basis of their impairments and bodily differences from the norm.

This finding – of normalization and minimization – speaks to the participants’ needing to navigate difficult material circumstances and a set of intersecting beliefs about relationships, women, and disability which create a sense that suboptimal or harmful circumstances are ‘the best that can be expected’. Disability is a risk factor that intersects with gender and poverty, making women with disabilities triply vulnerable to abuse and violence. These women are silenced by patriarchal ideologies constructing how women should perform their gendered roles in marriage or sexual partnerships, by forced dependence on their intimate partners, and by social stigma and isolation.

## Conclusion

IPV against women with disabilities is an under-researched but prevalent social problem that needs to be recognised and addressed by all stakeholders. Although women with disabilities urgently require supports to decrease their vulnerability, the role of perpetrator-related characteristics drawn from feminist disability and evolutionary psychology theories indicate the simultaneous need for a more sustained focus on perpetrators. Efforts need to be made to reduce patriarchal domination and male sexually proprietary behaviours, particularly in contexts where potential perpetrators are triggered by the vulnerability and the dependence of partners with disabilities (Brownridge, 2006). Men who support patriarchal power differentials in relationships with women with disabilities need to receive the message that these ideologies, and the construction of gendered identities which render women vulnerable to exploitation and abuse, will be met with intervention that empowers both men and women with disabilities to engage in meaningful and equitable partnerships (Brownridge, 2006). At the same time, women with disabilities may sometimes need to be educated about what is ‘normal’ and acceptable in intimate partnerships. Accessible support services may be key to assisting these women to assert their rights, and it may also be the case that access to alternative forms of housing and income may be key drivers to give these women meaningful and sustainable alternatives to living with abusers. The problem of violence and abuse, though, is essentially a problem of perpetration. It is only through a dual focus on both men and their partners with disabilities, that the women with disabilities may be protected from violence and exploitation.

Service providers working with women with disabilities need to acknowledge the vulnerability women with disabilities face, and there should be efforts to expand programming to involve screening, awareness raising and educational activities which develop and enhance skills and strategies that empower women to protect themselves. Capacity-development may also be needed among service providers to understand their own potential prejudices in relation to sexuality and disability and ensure that their own ability to work with women with physical disabilities is strengthened (Goyal, 2017). This needs to include facilitating access to necessary legal and protection services where there have been instances of abuse. Programmes should additionally extend community outreach programming to educate and raise awareness among potential perpetrators (Nosek et al., 2001). These interventions need to focus on the role of patriarchy in constructing gender identities, address power differentials in relationships, and offer support and skills to those partners who are also assuming caregiving roles.
